# Imaging real-time HIV-1 virion fusion with FRET-based biosensors

**DOI:** 10.1038/srep13449

**Published:** 2015-08-24

**Authors:** Daniel M. Jones, Sergi Padilla-Parra

**Affiliations:** 1 Division of Structural Biology, University of Oxford, The Henry Wellcome Building for Genomic Medicine, Headington, Oxford OX3 7BN, UK; 2 Cellular Imaging Core, Wellcome Trust Centre for Human Genetics, University of Oxford, Oxford, UK

## Abstract

We have produced a novel, simple and rapid method utilising genetically encodable FRET-based biosensors to permit the detection of HIV-1 virion fusion in living cells. These biosensors show high sensitivity both spatially and temporally, and allow the real-time recovery of HIV-1 fusion kinetics in both single cells and cell populations simultaneously.

Prior to establishing a productive infection, HIV-1 must first enter a target cell, a process that requires attachment to cellular receptor CD4 and coreceptors CXCR4 or CCR5 followed by fusion between the viral lipid envelope and the cell membrane^1^. A number of assays are available to measure HIV-1 entry, including β-galactosidase expression^2^ electron microscopy and tomography^3^, measurement of intracellular p24 (viral capsid protein)^4^ and colocalization of viral antigens with cellular proteins using confocal microscopy^5^. These assays, however, cannot directly detect the moment of viral fusion in real time. To accomplish this, various entry-specific assays have been developed over the last decade and include the redistribution of an unquenching fluorophore embedded in the virus envelope^6^, the photosensitized activation of a hydrophobic dye loaded in the host membrane^7^, or more recently, real-time single virus tracking (SVT) techniques^8^. The latter method is very sensitive and permits the detection of single virus fusion events^9^ but is time consuming and technically difficult. The β-Lactamase (BlaM) assay represents a widely used technique for measuring viral fusion in a population of cells^10^. However, the BlaM assay has caveats which include the need to add fusion inhibitors (temperature block, chemicals or drugs) at various time points in order to recover kinetic data^11^,^12^. This necessity arises due to slow cleavage of the CCF2 substrate, preventing real-time data from being obtained. In addition, the BlaM assay is frequently combined with spinoculation (centrifugation of cells in the presence of virus). This technique may alter cell integrity and boost fusion beyond the natural capacity of the virus, enabling the use of low multiplicities of infection (MOI) that otherwise may not provide detectable levels of fusion^12^,^13^,^14^. Here, we propose a novel, simple and rapid assay allowing the real-time detection of HIV-1 fusion with living cells at both the single-cell and cell population level in the absence of spinoculation. Using this approach, the cells remain alive during HIV-1 entry and fusion as well as post-imaging, thereby permitting further screening and analysis to be performed. It is also highly scalable to high-throughput screening assays—one of which could be screening for small molecule fusion inhibitors.

## Results and Discussion

**Figure 1:**
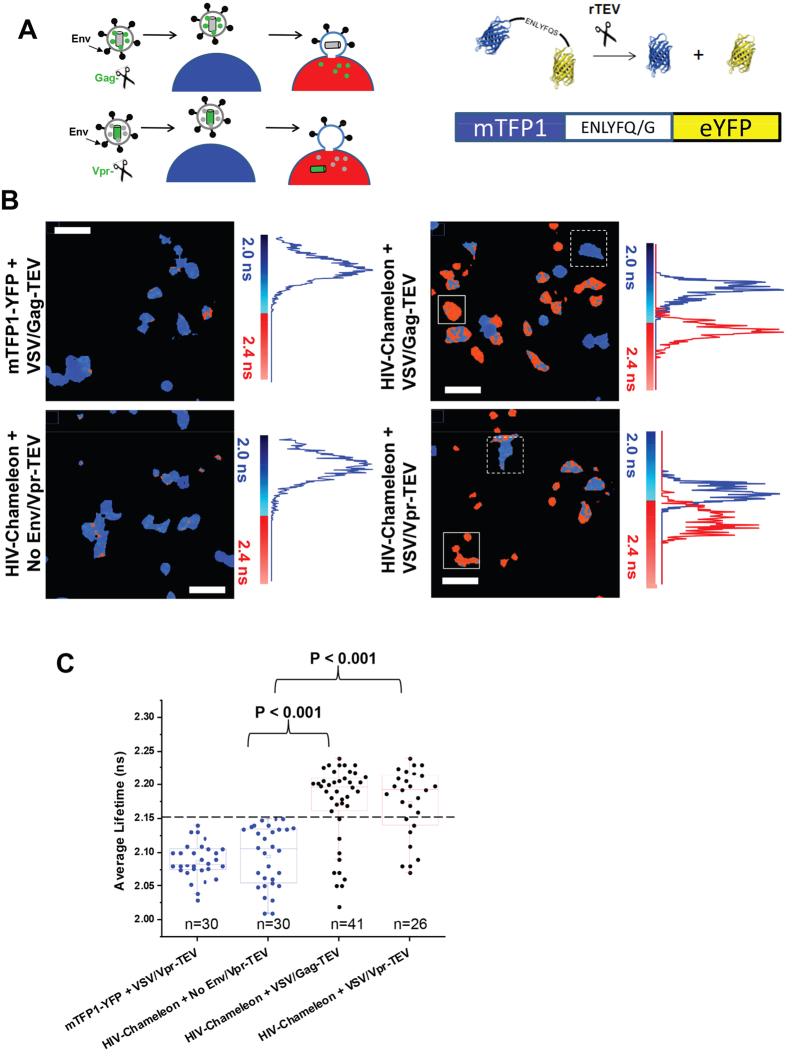
Transient expression of HIV-1-Chameleon in target cells permits sensitive detection of virus fusion. (**A**) Illustration of the HIV-chameleon based fusion assay. The TEV protease (TEVp) can be fused to either the HIV-1 Vpr protein or the Gag protein, and is incorporated into viral particles during packaging in both cases. Viruses are then added to target cells transfected with the HIV-Chameleon biosensor. When fusion occurs, Vpr-TEV or Gag-TEV are released into the cytosol and cleave the recognition sequence within HIV-Chameleon, disrupting FRET. The TEVp substrate sequence (ENLYFQ/G) found in the linker sequence between mTFP1 and eYFP is also shown. (**B**) FLIM pseudocolour images are presented alongside the histogram of lifetimes for all pixels in the image, where blue indicates lower lifetimes (no fusion) and red represents higher lifetimes (fusion). TZM-bl cells expressing a tandem lacking the TEVp recognition site (mTFP1-eYFP) were incubated with virions harbouring Gag-TEV and pseudotyped with envelope proteins from VSV (top left panel) as a control. Cells expressing HIV-Chameleon were incubated with No Env/Vpr-TEV (bottom left panel), VSV/Gag-TEV (top right panel) and VSV/Vpr-TEV (bottom right panel). FLIM experiments were performed using the 20X magnification objective. Scale bar = 30 μm. In the right panels, dashed regions of interest (ROIs) highlight non-fusogenic cells (blue) whilst the solid squares show fusogenic cells (red). (**C**) A box-plot representing the mean lifetimes derived from individual cells is shown, and highlights statistically significant differences between cells treated with negative controls (blue boxes) or viruses (red boxes). Conditions are as follows: cells expressing mTFP1-YFP + VSV/Vpr-TEV (n = 30) and those expressing HIV-Chameleon + No Env/Vpr-TEV (n = 30), VSV/Gag-TEV (n = 41) or HIV-VSV/Vpr-TEV (n = 26). All error bars represent standard deviation of the mean value. Values above the dashed black line are considered to be representative of fusion, whereas those below represent non-fusogenic cells. All data was acquired from at least 3 separate experiments.

**Figure 2:**
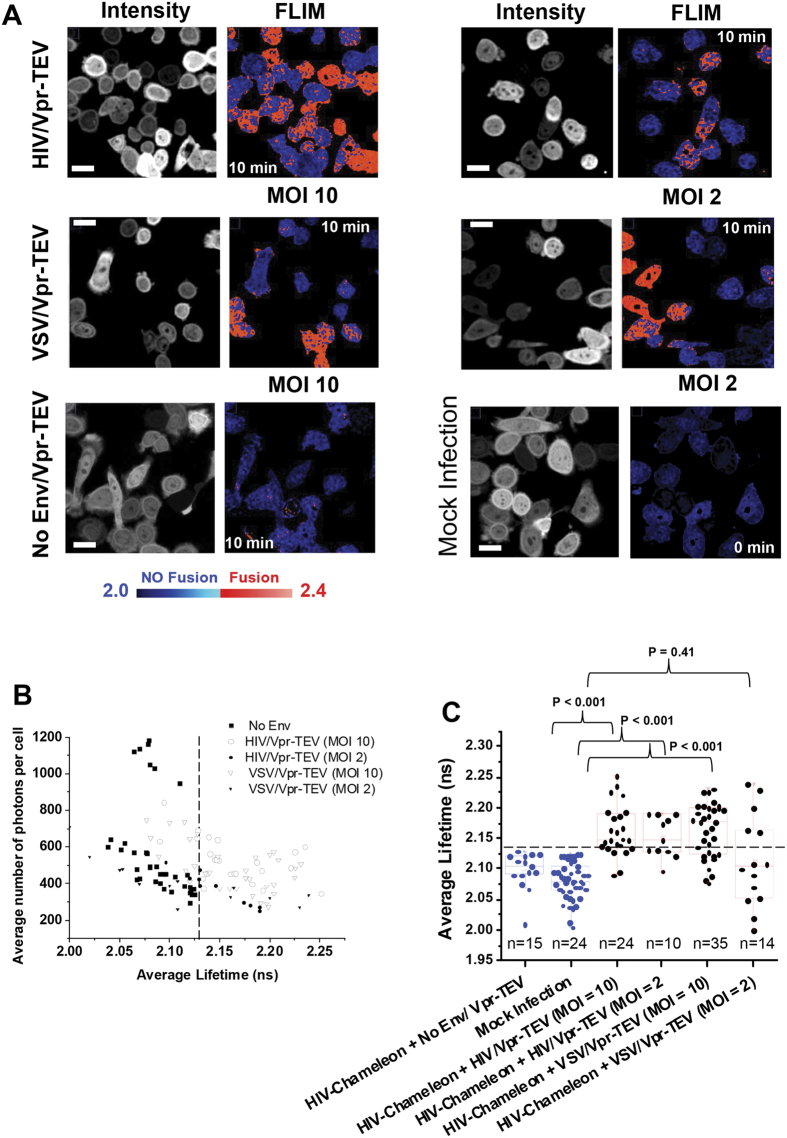
Stable expression of HIV-Chameleon combined with FRET-FLIM analysis permits sensitive detection of viral fusion. (**A**) TZM-bl cells stably expressing HIV-Chameleon were treated with HIV/Vpr-TEV (top panels) and VSV/Vpr-TEV (middle panels) using MOIs of 10 (left column) and 2 (right column). Cells receiving No Env/Vpr-TEV (bottom left panel) or mock infected cells (bottom right panel) are also shown. Longer lifetimes (indicative of fusion) are shown in red while shorter lifetimes (representing non-fusion) are in blue. FLIM experiments were performed using the 63X magnification objective. Scale bar = 10 μm. (**B**) A plot representing the mean lifetimes coming from individual cells versus the fluorescence intensity is depicted and shows that lifetime values are independent of the level of HIV-Chameleon expression. Values to the left of the dashed black line represent cells where fusion did not occur, whereas those to the right represent fusion-positive cells (**C**) A box-plot representing the mean lifetimes of fusogenic and non-fusogenic cells is shown, and highlights statistically significant differences between control-treated (blue boxes) or virus-treated (red boxes) cells. Conditions are as follows: HIV-Chameleon + NoEnv/Vpr-TEV (n = 15), mock infected cells (n = 24), and HIV-Chameleon + HIV/Vpr-TEV at MOI 10 (n = 24) and MOI 2 (n = 10) or VSV/Vpr-TEV at MOI 10 (n = 35) and MOI 2 (n = 14). Error bars represent standard deviation of the mean. Values above the dashed black line are considered to be representative of fusion, whereas those below represent non-fusion. All data was acquired from at least 3 separate experiments.

Our protease/substrate system (Fig. 1A), relies on the expression of a genetically-encodable fluorescent biosensor (termed HIV-Chameleon) within target cells. The biosensor is comprised of a pair of fluorophores (mTFP1 and eYFP) capable of undergoing FRET and held together by a short peptide sequence containing the Tobacco Etch Virus (TEV) protease (TEVp) cleavage site^15^. When biosensor-expressing cells are exposed to viruses encapsulating the TEV protease, productive fusion results in cleavage of the biosensor and an altered FRET profile that can be quantified in real-time. We first tested the best approach for incorporating TEVp into virus particles; by fusing TEVp (i) to HIV-1 Vpr, which is packaged within the viral nucleocapsid (Vpr-TEV) and (ii) between the matrix and capsid proteins of HIV-1 Gag (Gag-TEV). For the initial test, HIV-1 particles were pseudotyped with the well characterised envelope G protein from vesicular stomatitis virus (VSV). These pseudoviruses were then added to the transfected cells at an MOI of 10 and the donor lifetime of the biosensor (mTFP1) was measured by fluorescence lifetime imaging microscopy (FLIM), an established technique particularly well suited for mTFP1-YFP couples^16^,^17^. Cells receiving both VSV/Vpr-TEV and VSV/Gag-TEV displayed increased mean lifetimes as compared to the mean FRET lifetime of cells where the mTFP1-eYFP substrate did not harbour the TEV cleavage site (Fig. 1B,C), or to cells receiving particles encoding no envelope proteins (No Env/Vpr-TEV). When comparing these cells against cells expressing HIV-Chameleon treated with both VSV/Vpr-TEV and VSV/Gag-TEV, statistically significant differences were found (P < 0.001 in both cases). It should be noted that the threshold for fusion was defined by the uppermost values obtained from cells treated with No Env/Vpr-TEV, and that each condition produced a spectrum of fusion-positive and fusion-negative phenotypes. Since we found little difference between TEV tagging methods, the Vpr-TEV approach was used for all further experimentation. We next produced a cell line that continuously and stably expressed the HIV-Chameleon biosensor. This was a beneficial approach since it eliminated the need for transfection. Additionally, levels of HIV-Chameleon expression across the cell population were similar and near 100% of the cells were positive for biosensor expression. The HIV-Chameleon cell line was infected with both VSV- and HIV- (strain JR-FL) pseudotyped particles at MOIs of 10 and 2 (Fig. 2). Cells receiving both VSV/Vpr-TEV and HIV/Vpr-TEV displayed increased mean lifetimes as compared to those from cells receiving No Env/Vpr-TEV (P < 0.001) or mock infected cells. For cells receiving VSV at a MOI of 2, fusogenic cells were observed but their numbers were fewer, meaning that the mean lifetime was ultimately pulled below the fusion threshold (Fig. 2C, P = 0.41). Overall though, increased lifetimes correlated directly with TEV protease activity and, by implication, virus fusion. To further prove the specificity of our biosensor, the HIV-Chameleon cell lines was inoculated with VSV/Vpr-TEV or HIV/Vpr-TEV (MOI 10) in the presence of Dynasore (a widely used dynamin inhibitor^18^) to prevent fusion occurring (Supplementary Fig. 1). Lifetime values coming from HIV-Chameleon in Dynasore-treated cells remained unperturbed in the presence of virus, demonstrating that genuine fusion events were required for HIV-Chameleon cleavage. As expected, all FLIM measurements were independent of the level of biosensor expression as long as the average numbers of photons for cells analysed were kept between 250 and 1200 (Fig. 2B).

**Figure 3:**
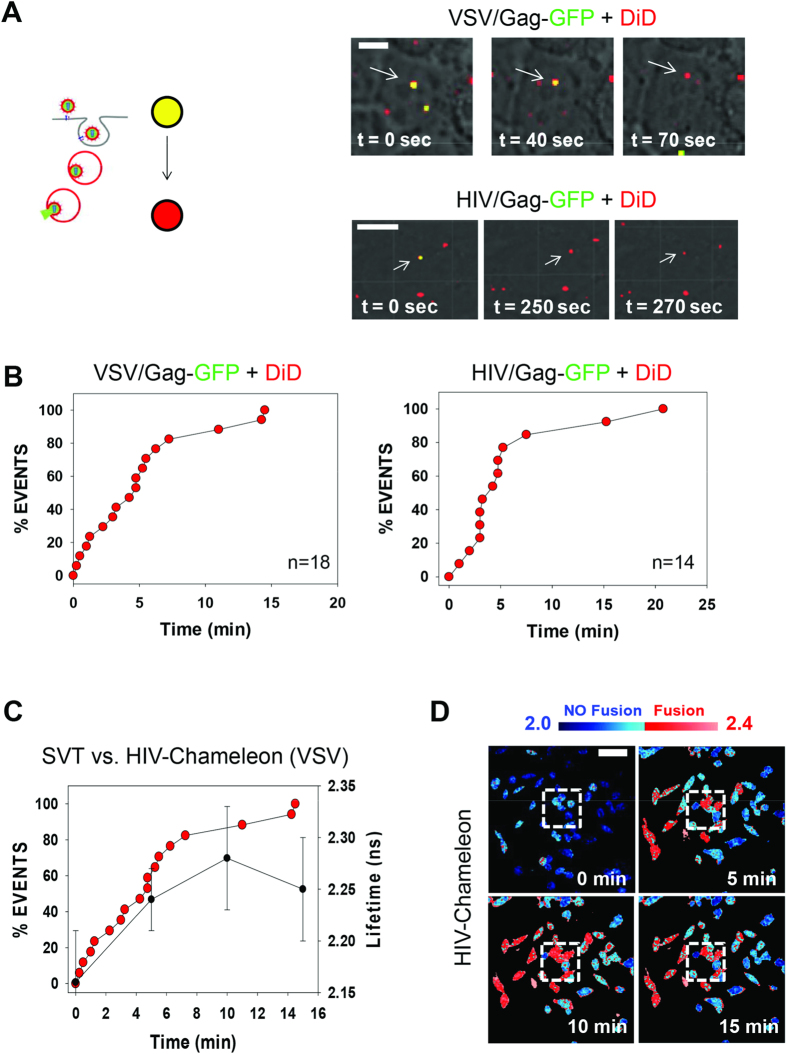
Real-Time Single Virus Tracking (SVT) of double-labelled virus particles. (**A**) A cartoon outlining how double labelled particles can be employed to identify virus fusion—DiD (red) redistributes within the endosomal membrane during fusion and remains visible whilst the Gag-GFP (green) signal is lost. Thus, the time at which the green signal disappears represents the time fusion occurred. (**B**) Double-labelled virus particles pseudotyped with envelope proteins from VSV (left panel, n=18) or HIV (right panel, n = 14) were added to living TZM-bl cells. SVT was performed only on viruses labelled with both colours and typical time-lapse series for both viruses are shown. Experiments were performed using 63X magnification. Scale bar = 5 μm. (**C**) a graph depicting VSV fusion rates, as measured by SVT (red points, measured as % events) compared to using the HIV-Chameleon cell line (black points, measured as lifetime in nanoseconds). Error bars represent standard deviation of the mean value in each case. (**D**) Images from which the fusion data in Fig. 3C were derived. Lifetime measurements were calculated from the cells within the region of interest (white dashed box).

We next performed several SVT experiments in living cells in order to compare the fusion kinetics obtained to those gathered from the HIV-Chameleon cell line. For these experiments, HIV-based pseudoparticles with VSV or HIV envelopes were double-labelled through incorporation of a Gag-GFP fusion protein and the lipophilic dye DiD^12^. Such labelling strategies have been used previously to demonstrate that single HIV-1 virions fuse within endosomes in TZM-bl cells and T-derived cells^12^,^13^. It should be noted that these viruses did not harbour the Vpr-TEV fusion protein. For both VSV- and HIV- pseudotyped viruses, the Gag-GFP content marker was lost whilst the membrane marker (DiD) remained (Fig. 3A), indicating that fusion likely occurred within endosomes for both viruses. We plotted the cumulative distribution of these events for both VSV and HIV pseudotyped virions (Fig. 3B). The majority of VSV- and HIV-fusion events occurred ~10–15 minutes after virus addition (Fig. 3B). It is important to note that SVT measurements differ substantially to those obtained using lifetime imaging; for SVT, each point on the graph represents a single virus fusion event and a cumulative distribution arising from multiple experiments is then applied to recover the overall fusion kinetics. By comparison, lifetime measurements (such as those gained using HIV-Chameleon) represent the average score obtained from a number of cells or individual cells within a defined region of interest. Despite these differences, the two methods displayed remarkable similarity in fusion rate, as shown using VSV as an example (Fig. 3C). Thus, the HIV-Chameleon cell line is capable of yielding fusion data with sensitivity comparable to that obtained from SVT techniques.

**Figure 4:**
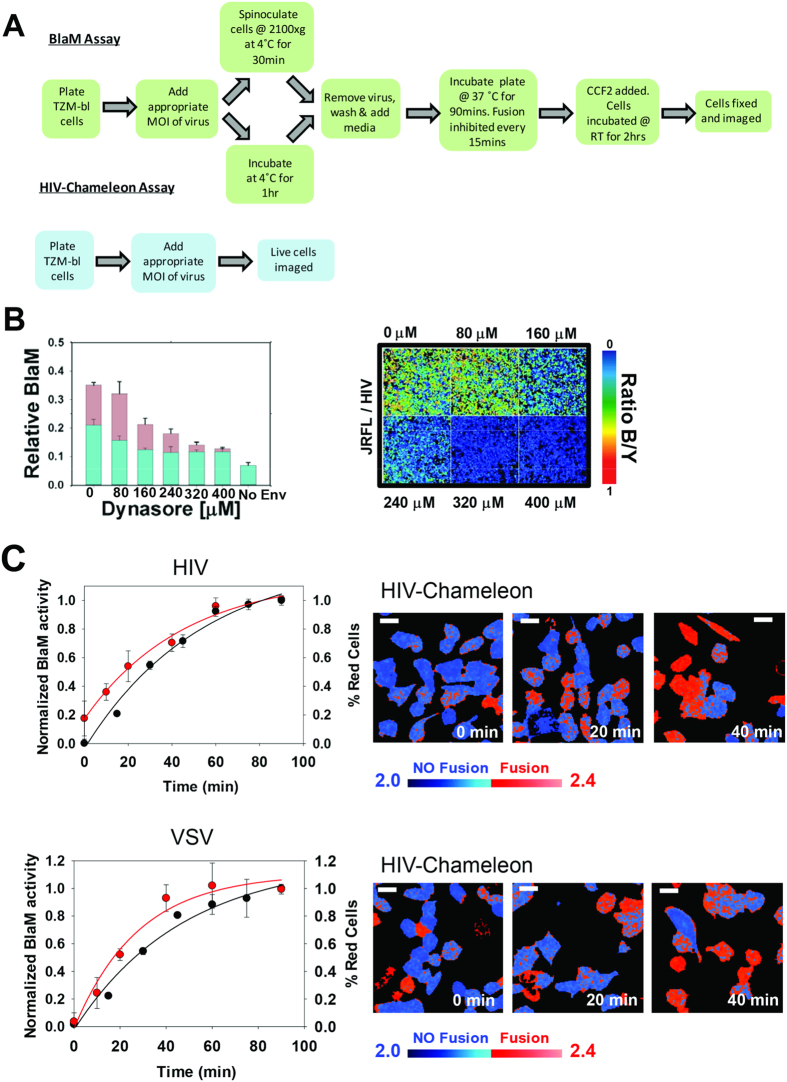
Comparison between the HIV-Chameleon system and the BlaM assay for measurement of virus fusion across a population of cells. (**A**) A schematic comparing the steps involved for the BlaM and HIV-Chameleon assays to measure viral fusion. For the BlaM assay, cells are plated in a 96-well plate (1 well per time point to be measured) for 24 hrs prior to exposure to virus. Cells with virus are either spinoculated or allowed to interact in the absence of centrifugal force. In both cases, cells are maintained at 4 °C to prevent virus internalisation. Subsequently, cells are washed with PBS before receiving fresh medium and being shifted to 37 °C to initiate virus internalisation using a time of addition approach. A kinetic curve can then be constructed. For the HIV-Chameleon assay, cells autonomously expressing the cleavable biosensor are plated in the same fashion as the BlaM assay. 24 hours later, cells can be immediately placed into the 37 °C incubator located on the microscope. Virus is then added and the blue/yellow ratio is measured during the virus internalisation process. Since a time-of-addition approach is not required here, kinetics are measured in real time within living cells. (**B**) A titration experiment was used to identify the concentration of Dynasore required to prevent fusion for VSV/Vpr-BlaM (cyan bars) and HIV/Vpr-BlaM (pink bars). 400 μM Dynasore was picked as the most appropriate concentration to use, since it decreased fusion levels for both viruses to levels comparable to those seen with the No Env/Vpr-BlaM control. An example of the images used to quantify the decrease in viral fusion is shown on the right. (**C**) A comparison between fusion kinetics obtained with the BlaM assay (black lines, measured as normalised BlaM activity) and the HIV-Chameleon cell line (red lines, measured as % red cells). Examples of the type of image (only 0, 20 and 40 minutes are shown) recovered from the HIV-Chameleon cell line and used to quantify the level of fusion are shown to the right of each graph, and clearly show cells in which viral fusion has taken place (red cells). Error bars represent standard deviations. An MOI of 10 was used for both assays (HIV-Chameleon and BlaM) to ensure kinetics were derived from comparable amounts of input virus.

Finally, we sought to compare the HIV-Chameleon system with the widely used BlaM assay. Firstly, it is noteworthy that a far more complex protocol was required for the BlaM assay (Fig. 4A), since fusion kinetics can only be obtained through a time-of-addition approach where fusion is halted at pre-chosen time points. For these experiments, we used 400 μM Dynasore to block fusion at 15, 30, 45, 60, 75 and 90 minutes after fusion was initiated. A titration experiment was used to choose the appropriate concentration of Dynasore (Fig. 4B). Cells also needed to be fixed to prevent leakage of the CCF2 substrate and cleavage products^19^. By comparison, the HIV-Chameleon system involved the addition of virus particles to cells followed by direct imaging—cells remained live throughout the procedure and no Dynasore treatment was required. To recover kinetics from multiple cells at once from the HIV-Chameleon cell line, the percentage of red (fusion-positive) cells as a function of time was recorded (Fig. 4C). A plateau in the fusion kinetics curve was attained after 40 and 60 minutes (red line in Fig. 4C) for HIV/Vpr-TEV (top panel) and VSV/Vpr-TEV (bottom panel) respectively. For the BlaM assay, fusion times were remarkably similar to those recorded using HIV-Chameleon with both viruses (black lines, Fig. 4C). Thus, the sensitivity gained by combining FRET-FLIM with the HIV-Chameleon biosensor permitted us to follow fusion kinetics in a single population of living cells in real time and importantly, HIV and VSV fusion matched that seen using the widely-used BlaM assay. Critically, data was gained in the absence of spinoculation, which dramatically enhanced the level of virus fusion across a range of MOIs when using the BlaM assay (Supplementary Fig. 2).

In summary, the assay described here offers a more streamlined protocol than the widely-used BlaM assay and eliminates the need for tedious time-of-addition approaches, meaning fusion kinetics can be obtained in real time. We show that fusion measurements are comparable to those obtained using SVT, currently regarded as the most accurate system for measuring viral fusion events. It should be noted however that unlike SVT, the HIV-Chameleon assay measures events occurring within single cells rather than events arising from single virus particles. However, this allows the user some flexibility in terms of whether they wish to study single cells or a wider population of cells—in fact, both can be achieved using HIV-Chameleon. One caveat is that the assay is currently incompatible with T-cell lines and primary human T cells due to the requirement for transfection/cell line production. Despite this limitation, the system proposed here could be easily adapted for high-throughput screening approaches that might identify inhibitors of virus entry mechanisms that could then be confirmed in primary cells. The system would also be relevant for improving our understanding of the cellular determinants required for virus entry in general, and could be applied to any virus that effectively lend their envelope proteins to the pseudoparticle system, such as Dengue, Ebola or HCV.

## Materials & Methods

### Plasmids

pR8ΔEnv (encoding the HIV-1 genome harbouring a deletion within Env), pcRev, Gag-GFP, H1N1 and VSV-G were kindly provided by Greg Melikyan (Emory University, Atlanta, USA). The plasmid encoding the JR-FL envelope protein was a kind gift from James Binley (Torrey Pines Institute for Molecular Studies, California, USA).

### Cell culture

HEK-293T cells and TZM-bl cells were grown using Dulbecco’s Modified Eagle Medium (DMEM; Life Technolgies) supplemented with 10% foetal bovine serum, 1% Penicillin-Streptomycin and 1% L-Glutamine to give DMEM complete (DMEM_comp_). All cells were maintained in a 37 °C incubator supplied with 5% CO_2_.

### PCR

Reactions were performed using the TaKaRa Pyrobest DNA polymerase kit (Clontech). Reactions typically contained ~50 ng DNA template, 10 μM forward and reverse primers, dNTPs (2.5 mM each), 1x Pyrobest buffer and 5 units of Pyrobest DNA polymerase. Reactions were made up to 50 μl using water. PCR was performed in a Veriti 96-well Thermal Cycler using the following steps: (i) denaturation of DNA at 95 °C for 2 mins (ii) strand separation at 95 °C for 30 seconds (iii) primer annealing at 55 °C for 30 seconds (iv) strand elongation at 72 °C for 1 minute/Kb of DNA. Steps (ii)–(iv) were repeated 34 times. PCR products were run on a 1% agarose gel prior to purification using the QIAquick Gel Extraction System (Qiagen) in accordance with the manufacturer’s instructions.

### Cloning

To produce the HIV-Chameleon biosensor, mTFP1-eYFP was used as a template for the production of a 788 bp fragment termed mTFP1-TEV, which consisted of the mTFP1 ORF plus a C-terminal peptide spacer harbouring the TEV protease recognition sequence. PCRwas performed using forward primer 5′-AGATCCGCTAGCGCTACCGGTCGC-3′ (incorporating an existing *BmtI* site) and reverse primer 5′-AGATCGGATCCGGACCTTGAAAATAAAGATTTTCTCGAGATCTGAGTCCGGACTTGTACAG-3′ (introducing the TEV substrate sequence and a *BamHI* site). The mTFP1-TEV PCR fragment was then digested with *BmtI* and *BamHI* and introduced back into mTFP1-eYFP digested with the same enzymes to create HIV-Chameleon. To create Vpr-TEV, pRK793 (obtained from Addgene) was used as a template for the amplification of the 742 bp catalytic domain of the TEV protease domain. Forward primer 5′-GATCTGCTAGCATGGGAGAAAGCTTGTTTAAGGGG-3′ (introducing an N-terminal *BmtI* site) and reverse primer 5′-AGATCCTCGAGATCTGAGTAATTCATGAGTTGAGTCGCTTC-3′ (introducing a C-terminal *XhoI* site) were used for the PCR amplification of this sequence. Subsequently, the fragment was digested with *BmtI* and *XhoI* and ligated into a Vpr-YFP plasmid digested in the same manner. This strategy resulted in the replacement of YFP with the catalytic domain of the TEV protease, producing Vpr-TEV. To generate Gag-TEV, a similar strategy to the production of Vpr-TEV was utilised. Again, pRK793 was used as a template to amplify the same catalytic region of the TEV protease. This time, the product was 730 bp and flanked by *MluI* (N-terminal) and *XbaI* (C-terminal) sites, produced by using forward primer 5′-GATCTACGCGTGGAGAAAGCTTGTTTAAGGGG-3′ and reverse primer 5′-AGATCTCTAGAATTCATGAGTTGAGTCGCTTC-3′. The PCR product was digested with *MluI* and *XbaI* before being ligated into a Gag-mCherry backbone plasmid digested with the same enzymes. This resulted in Gag-TEV, where the TEV protease is located between the HIV matrix and capsid sequences. More details on cloning all cloning protocols are available upon request. All constructs were verified by sequencing.

### Generation of the HIV-Chameleon stable cell line

All cloning related to cell line generation was performed using the Gateway cloning system (Life Technologies). First, the TZM-Chameleon biosensor was amplified by PCR using forward primer 5′- GGGGACAAGTTTGTACAAAAAAGCAGGCTTCCCGTCAGATCCGCTAGCGC-3′ and reverse primer 5′-GGGGACCACTTTGTACAAGAAAGCTGGGTCGGCTGATTATGATCTAGAGTC-3′, thereby flanking the product with attB1 recombination sites. This product was then introduced into the pENTR.221 entry plasmid by way of BP recombination (in accordance with manufacturer’s instructions). The HIV-Chameleon sequence was then flipped into the final destination plasmid, pHAGE-N-HA-FLAG, by way of LR recombination to create pHAGE-HIV-Chameleon. Next, lentiviruses packaging the biosensor were produced by transfecting HEK 293T cells with the following components: 8 μg pHAGE-HIV-Chameleon, 0.4 μg Gag-Pol, 0.4 μg Rev, 0.4 μg Tat and 0.8 μg VSV-G. For further details on transfection procedure, please refer to the ‘virus production’ section of the materials and methods. Supernatants harbouring virus particles were harvested 48 hrs post-infection and 6 ml were used to infect TZM-bl cells at 50% confluency in a T75 flask. 24 hrs later, the supernatants were removed, cells were washed with PBS, and 10 ml of DMEM_comp_ containing 2 μg/ml Puromycin was added. Cells were thereafter passaged and cultured in the presence of Puromycin and integration of the biosensor was confirmed by fluorescence microscopy.

### Virus production

Pseudotyped viral particles were produced by transfecting HEK-293T cells plated at ~60–70% confluency in T75 or T175 flasks. DNA components were transfected using GeneJuice (Novagen) in accordance with the manufacturer’s instructions. To produce particles harbouring the TEV protease, cells were transfected with 2 μg pR8ΔEnv, 2 μg Vpr-TEV, 1 μg pcREV and 3 μg of the appropriate viral envelope (either VSV-G or the CCR5-tropic HIV-1 strain JR-FL). For viruses harbouring Gag-TEV, 2 μg Vpr-TEV was substituted for 3 μg of the Gag-TEV plasmid. Similarly, 2 μg of Vpr-BlaM was used instead of Vpr-TEV for viruses compatible with the BlaM assay. Transfection mixtures were then added to cells in DMEM_comp_ before returning flasks to the 37 °C CO_2_ incubator. 12 hrs post-transfection, the transfection mixture-containing medium was removed and cells were washed with PBS. Fresh DMEM_comp_ (lacking phenol red) was then added. Cells were subsequently incubated for a further 24 hrs. 48 hrs post-transfection, viral supernatants were removed from cells and pushed through a 0.45 μm syringe filter (Sartorius Stedim Biotech) before being aliquoted and stored at −80 °C. For SVT-compatible virus production, cells were transfected in the same manner with 2 μg pR8ΔEnv, 3 μg Gag-GFP, 1 μg pcREV and 3 μg of the appropriate viral envelope (either VSV-G or JR-FL). 12 hrs post-transfection, the transfection complexes were removed and cells were washed with PBS before being incubated at 37 °C with 10 ml Optimem (Life Technologies) containing 10 μM DiD (Life Technologies) for 4 hrs. Subsequently, the staining mixture was removed, cells washed twice with PBS and fresh DMEM_comp_ (lacking phenol red) was then added. Cells were incubated for a further 24 hrs prior to harvesting.

### BlaM assay

24 hrs prior to the assay, TZM-bl cells were plated at 4 × 10^4^ cells/well in black clear-bottomed 96 well plates. On the day of assay, cells were cooled on ice prior to the addition of the appropriate MOI of virus (all infections were performed in 100 μl volumes). Immediately following addition of virus harbouring Vpr-BlaM, cells were either [i] centrifuged at 2100×g for 30 mins at 4 °C or [ii] placed at 4 °C for 1 hr. Virus was then removed and cells were washed with PBS, and 100 μl of DMEM_comp_ was added to each well before shifting the plate to the 37 °C CO_2_ incubator to initiate viral entry. To gain kinetic data, virus fusion was blocked at the appropriate time point (0, 15, 30, 45, 60, 75 and 90 mins) by removing the media and replacing with media containing Dynasore (Sigma-Aldrich). The inhibitor concentration of Dynasore (400 μM) was found by testing different concentrations in a titration experiment (Fig. 4). Note that for the 0 min time point, Dynasore was added immediately prior to the 37 °C temperature shift. After 90 mins, cells were loaded with CCF2-AM from the LiveBLAzer FRET—B/G Loading Kit (Life Technologies) and incubated at room temperature in the dark for 2 hrs. Finally, the CCF2 was removed; cells were washed with PBS and fixed with 2% PFA prior to viewing.

### BlaM assay spectral analysis

TZM-bl cells loaded with CCF2 were excited using a 405 nm continuous laser (Leica, Manheim) and the emission spectra between 430–560 nm was recorded pixel by pixel (512 × 512) using a Leica SP8 X-SMD microscope with a lambda resolution of 12 nm. The ratio of blue emission (440–480 nm, cleaved CCF2) to green (500–540 nm, un-cleaved CCF2) was then calculated pixel by pixel using ImageJ (http://imagej.nih.gov/ij/) for three different observation fields using a 20X objective and plotted as a function of time. Fusion kinetics were then recovered from the time-stack of blue/green ratio and normalized to the maximum intensity. Two different mathematical models were used to fit the global fusion kinetics, an exponential growth model and a logistic growth depending on the shape of the curve. Fits were performed with Sigma Plot (San Jose, CA).

### Förster Energy Transfer by Fluorescence lifetime imaging microscopy (FRET-FLIM)

Living cells expressing HIV-chameleon were imaged before and after virion addition using a SP8–X-SMD Leica microscope from Leica Microsystems (Manheim, Germany). Areas of interest were chosen under either a 20x air immersion objective or a 63×/1.4 oil immersion objective. Cells were excited using a 440 nm pulsed laser tuned at 40 MHz coupled with single photon counting electronics (PicoHarp 300) and subsequently detected by Hybrid external detectors. To rule out artefacts due to photo–bleaching and insufficient signal to noise, only cells with at least 250–1000 photons per pixel and negligible amount of bleaching were included in the analysis after a 2 × 2 image binning^16^,^20^. The acquired fluorescence decay, of each pixel in one whole cell were deconvoluted with the instrument response function (IRF) and fitted by a Marquandt nonlinear least–square algorithm with one or two–exponential theoretical models using Symphotime software from Picoquant GmbH. The mean fluorescence lifetime (Tau) and fraction of interacting donor (f_D_) were calculated as previously described^17^,^20^ using Symphotime, Mapi software^20^ and ImageJ (http://imagej.nih.gov/ij/). Statistical analysis of the lifetime data was performed using a two-tailed t-test or Rank Sum test (SigmaPlot, San Jose, CA).

### Imaging virus entry and fusion

TZM-bl cells transiently or continuously expressing HIV-Chameleon plated in DMEM_comp_ lacking phenol red were placed into the microscope for examination. After selecting a region of interest, medium was removed from the cells and replaced with medium containing virus particles at the appropriate MOI. Fluorescence lifetime was then measured as described above. A threshold for fusion (as indicated by dashed black lines in Figs 1C and 2C,D) was established by measuring the lifetimes of cells receiving No Env/Vpr-TEV. Therefore, any cells displaying lifetimes greater than the highest values seen with No Env/Vpr-TEV were deemed fusion-positive. For SVT experiments, TZM-bl cells were incubated with the appropriate MOI of double-labelled (Gag-GFP and DiD) for 30 minutes at 4 °C. Following this, supernatants were removed and the cells were washed with PBS to remove unbound virions. Cells were then mounted onto a microscope stage maintained at 37 °C. Once a suitable image field was chosen, virus internalization was initiated by adding 1 ml of warm HBSS and imaging commenced using a Leica SP8 confocal microscope with a 63×/1.4 NA oil immersion objective and 2 HyD internal detectors capable of single photon counting. Images were acquired every 8–12 sec for ~45 min. The axial position of a specimen during acquisition was stabilized using the Adaptative Focus module. GFP was excited at 488 nm and emitted light was collected at 500–550 nm. DiD was excited at 633 nm and the emission range was 640–700 nm. These imaging conditions ensured negligible bleed through between GFP and DiD channels. Virus particles that entered the cell and fused were tracked using the red channel, whereas those undergoing hemifusion were tracked using the green channel as in^21^. Real time single particle tracking analyses yielded the mean fluorescence intensity of viral particles for both channels red and green, as well as their coordinates, as a function of time. SVT analysis was performed using Imaris software (BitPlane, Switzerland). Analysis of the kinetic data was performed using a two-tailed t-test or Rank Sum test (SigmaPlot, San Jose, CA).

## Additional Information

**How to cite this article**: Jones, D.M. and Padilla-Parra, S. Imaging real-time HIV-1 virion fusion with FRET-based biosensors. *Sci. Rep.* **5**, 13449; doi: 10.1038/srep13449 (2015).

## Supplementary Material

Supplementary Information
