# Supplementary material for: Imaging real-time HIV-1 virion fusion with FRET-based biosensors
Source: Sci Rep. 2015 Aug 24;5:13449. doi: 10.1038/srep13449 (PMC4547133; doi:10.1038/srep13449)
Supplement: Supplementary Information [file srep13449-s1.pdf]

SUPPLEMENTARY INFORMATION

Imaging real-time HIV-1 virion fusion with FRET-based biosensors

Daniel M. Jones and Sergi Padilla-Parra*

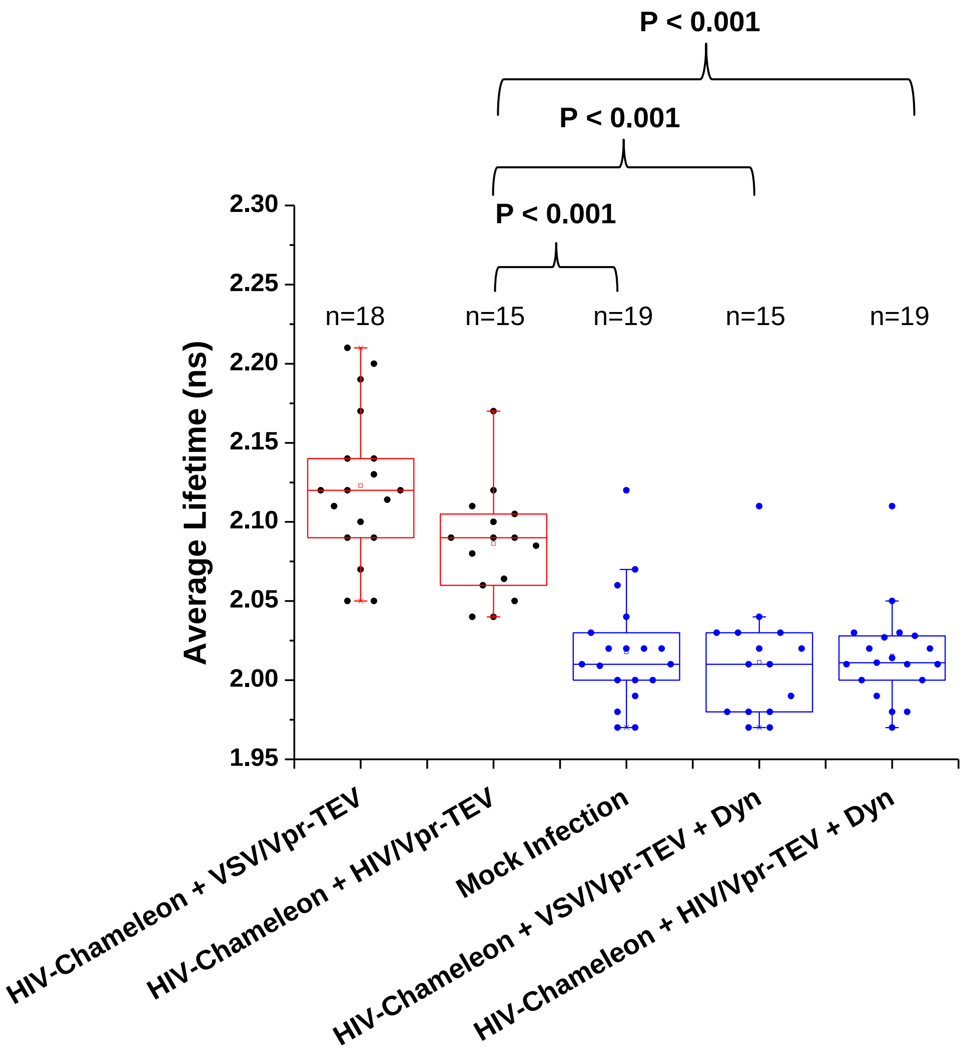

Supp Figure 1. Stable expression of HIV-Chameleon combined with FRET-FLIM analysis using virus-fusion inhibitors. TZM-bl cells stably expressing HIV-Chameleon were treated with HIV/Vpr-TEV or VSV/Vpr-TEV using MOIs of 10 (red boxes). Cells plated in parallel received the same virus treatments, this time combined with inhibitory concentrations of Dynasore (400µM, blue boxes). For comparison, the mean lifetime of mock infected cells is also shown. FLIM experiments were performed using the 63X magnification objective. All data was acquired from at least 3 separate experiments.

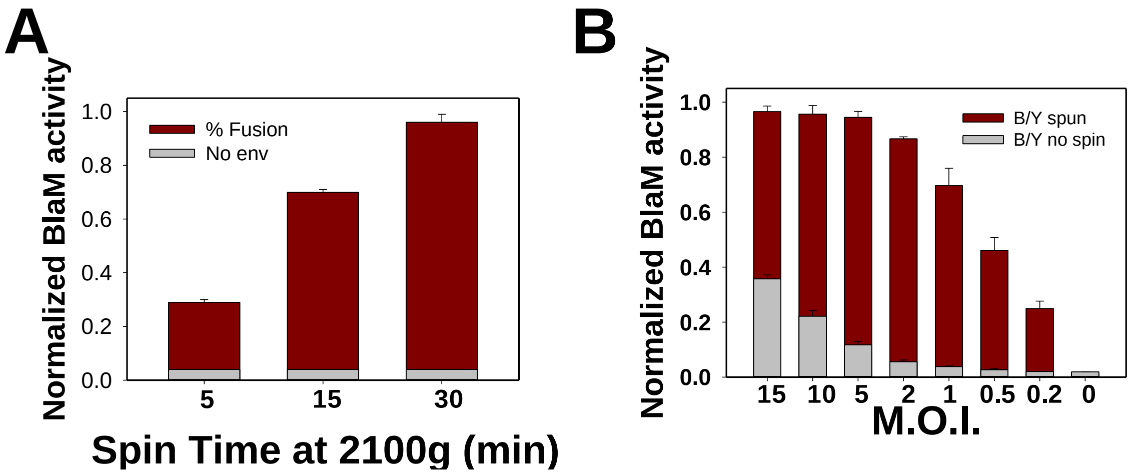

Supp Figure 2. Effects of spinoculation on virion fusion as measured using the BlaM assay. (A) VSV/Vpr-BlaM particles were introduced to TZM-bl cells at an MOI of 5 and at 4˚C to prevent virus internalisation prior to being centrifuged at 2100xg for 5, 15 or 30 minutes. BlaM readout indicated that longer spinoculation durations resulted in greater levels of fusion. (B) Comparison of fusion between spinoculated (B/Y spun) and non-spinoculated (B/Y no spin) TZM-bl cells across a range of MOIs. The extent of fusion was boosted by spinoculation for every MOI tested when compared to the No Env/Vpr-BlaM control. Increasing MOI also enhanced fusion, whether spinoculation was performed or not. It should be noted that for this experiment only, BlaM end point measurements were recovered using a CLARIOstar plate reader (and not the Leica SP8-X-SMD microscope as used elsewhere in the paper) in spectral mode with excitation at 405 nm and emission between 430 and 560 nm. This plate reader was not sufficiently sensitive to provide reliable data for lower MOIs (0.2, 0.5, 1 and 2) where cells were not spinoculated.
